# Oxidant-Free Electrochemical Direct Oxidative Benzyl Alcohols to Benzyl Aldehydes Using Three-Dimensional Printing PPAR Polyoxometalate

**DOI:** 10.3390/molecules28186460

**Published:** 2023-09-06

**Authors:** Wenhui Zhang, Ran Liu, Xueyan Lv, Lirong Jiang, Silu Tang, Gang Liu, Guodong Shen, Xianqiang Huang, Chen Ma, Bingchuan Yang

**Affiliations:** 1 School of Chemistry and Chemical Engineering, Liaocheng University, Liaocheng 252000, China; zwh2963105101@163.com (W.Z.); liuranbjt@163.com (R.L.); jlr17861826211@163.com (L.J.); tsl2766560274@163.com (S.T.); shenguodong@lcu.edu.cn (G.S.); hxq@lcu.edu.cn (X.H.); 2 School of Chemistry and Chemical Engineering, Shandong University, Jinan 250100, China; lxy17852267584@163.com; 3 College of Chemistry and Chemical Engineering, Qilu Normal University, Jinan 250013, China

**Keywords:** benzyl alcohol, benzaldehyde, electrocatalysis, polyoxometalate, 3D printing, additive manufacturing

## Abstract

The oxidation of benzyl alcohols is an important reaction in organic synthesis. Traditional methods for benzyl alcohol oxidation have not been widely utilized due to the use of significant amounts of precious metals and environmentally unfriendly reagents. In recent years, electrocatalytic oxidation has gained significant attention, particularly electrochemical anodic oxidation, which offers a sustainable alternative for oxidation without the need for external oxidants or reducing agents. Here, a copper monosubstituted phosphotungstate-based polyacrylate resins (Cu-LPOMs@PPAR) catalyst has been fabricated with immobilization and recyclability using 3D printing technology that can be successfully applied in the electrocatalytic oxidation of benzyl alcohol to benzaldehyde, achieving atom economy and reducing pollution. In this protocol, we obtain benzaldehyde in good yields with excellent functional group toleration under metal-free and oxidant-free conditions. This strategy could provide a new avenue for heterogeneous catalysts in application for enhancing the efficiency and selectivity of electrocatalytic oxidation processes.

## 1. Introduction

The oxidation of benzyl alcohols to aldehydes plays a key role in basic organic chemistry and organic industrial production [1]. The carbonyl compounds obtained by the oxidation of benzyl alcohols are widely used in natural products, agricultural chemistry, the fine chemical industry, and other fields [2,3,4,5]. Over the past few years, various strategies for oxidizing benzyl alcohols have been widely reported [6,7,8]. However, these methods mostly involved noble metals at high temperatures or even more severe conditions. At present, the traditional method to oxidize benzyl alcohols mainly uses KMnO_4_, K_2_CrO_4_, and MnO_2_ as the oxidant, and transition metals such as Pd, Ru, Os, Au, and Co as catalysts [9]. The traditional oxidation method has been met with limited success because of the use of noble metals and environmentally unfriendly reagents. Recently, some cleaning oxidants have also attracted considerable attention, such as H_2_O_2_ as terminal oxidants [10]. Briefly, although these oxidation reactions have been greatly improved in terms of being mild and environmentally friendly, there remains a need for the development of an economically viable, efficient, and straightforward catalytic system.

Organic electrochemical synthesis, a practical and environmentally friendly synthetic approach, has gained widespread attention worldwide for its extensive applications in oxidation, reduction, and redox-neutral reactions [11,12]. Electrochemical synthesis is an interdisciplinary field that combines organic chemistry with electrochemical techniques [13,14,15]. Organic electrocatalysis, as an efficient synthetic strategy, refers to the utilization of electricity to synthesize organic compounds, enabling redox transformations through anodic oxidation and cathodic reduction under conditions devoid of external oxidants or reductants [16,17]. From the perspective of circumventing the use of stoichiometric amounts of oxidizing or reducing agents, organic electrocatalysis undoubtedly represents a more environmentally friendly synthetic approach compared to traditional methods [18,19,20]. However, there is relatively limited research regarding the use of organic electrocatalysis for the oxidation of a series of benzyl alcohol compounds. The selection of an appropriate catalyst plays a crucial role in enhancing the efficiency and reducing the reaction time of electrochemical reactions [21,22,23,24,25]. Therefore, the development of an environmentally friendly and green electrocatalytic system with powerful heterogeneous catalysts holds significant scientific importance in achieving oxidative transformations without the use of oxidants or precious metal catalysts.

Polyoxometalates (POMs) are a class of metal–oxygen clusters spanning solute and solid metal oxide domains, composed of transition metals with the highest oxidation state (V, Nb, Ta, Mo, W) [26,27]. They have attracted rising attention on account of their super strong bronsted acidity, thermal stability, high proton mobility, and unique chemoselectivity [28,29]. POMs-based heterogeneous catalysts have been regarded as the environmentally friendly alternatives to familiar homogeneous acid catalysts [30]. However, the potential applications of polyoxometalates (POMs) as heterogeneous catalysts have been significantly limited due to their low specific surface area, challenging recyclability, and high solubility in polar solvents [31]. To conquer these issues, the design of immobilized polyoxometalates catalysts capable of facile recycling is highly desirable for electrocatalysis applications.

Three-dimensional printing technology, also known as additive manufacturing (AM), is a rapidly developing technique that combines digital model files and the layer-by-layer printing of different materials to achieve the rapid prototyping of target products [32,33,34]. Nowadays, 3D printing technology is being utilized more and more in the fields of agriculture, healthcare, and the automobile, locomotive, and aviation sectors for mass customization and manufacture [35]. One significant advantage of this technology is its ability to directly construct three-dimensional data models, providing possibilities for one-step rapid manufacturing of heterogeneous catalysts [36,37]. Currently, 3D printing technology has become a new research focus in the field of the one-step rapid manufacturing of heterogeneous catalytic materials [38,39,40]. Compared to traditional material processing techniques, 3D printing offers outstanding advantages such as digital manufacturing, personalized customization, environmental friendliness, and low energy consumption [41,42]. Therefore, utilizing 3D printing technology for the preparation of heterogeneous catalytic materials holds immense promise.

Herein, we have fabricated copper monosubstituted phosphotungstate-based polyacrylate resins (Cu-LPOMs@PPAR) using 3D printing technology, which can be successfully applied in the electrocatalytic oxidation of plenty of benzyl alcohol derivatives to corresponding metal- and oxidant-free aldehydes. Noteworthy, the thus-obtained Cu-LPOMs@PPAR possess remarkable stability, porous and loose architecture, well-dispersed Cu-LPOMs, shape designability, and adjustable loadings of Cu-LPOMs. Moreover, the immobilized catalyst can be easily detached and reused for six cycles without the significant loss of the catalyst. These results may provide a promising approach to prepare Cu-LPOMs@PPAR as a heterogeneous catalyst for metal- and oxidant-free electrochemical oxidation.

## 2. Results and Discussion

### 2.1. Performance Test of Cu-LPOMs@PPAR

**Figure 1:**
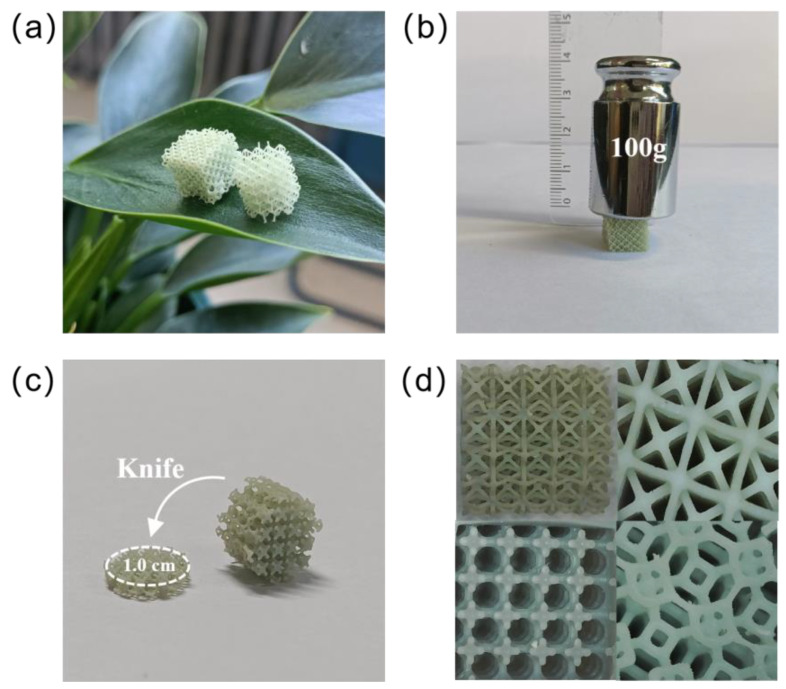
Physical properties of Cu-LPOMs@PPAR (30 wt%). (**a**) The photograph of the polyoxometalates catalyst standing on the leaf. (**b**) The photo image of the pressing experiment. (**c**) The photo image of cutting the catalyst into a fixed shape with a knife. (**d**) The pictures of catalysts taken by microscope.

To investigate the physical properties of Cu-LPOMs@PPAR (30 wt%), a series of relevant experiments were conducted. It was observed that the Cu-LPOMs@PPAR (30 wt%) sample could successfully stand on a leaf, indicating its low density and lightweight nature (Figure 1a). Additionally, Cu-LPOMs@PPAR (30 wt%) exhibited a tolerance for a weight of 100 g during a weight-pressing test (Figure 1b). Furthermore, the obtained catalyst could be easily sliced with a knife, making it readily fabricable and applicable in various experimental scenarios (Figure 1c), providing significant convenience. Microscopy images of Cu-LPOMs@PPAR (30 wt%) with shape designability demonstrated microstructures with different shapes that can be developed using 3D printing techniques, revealing a typical porous cellular texture with plentiful macropores (Figure 1d). These results collectively demonstrate that Cu-LPOMs@PPAR (30 wt%) possesses a loose structure with macropores, highlighting its potential as a porous heterogeneous catalyst for electrocatalytic oxidation.

**Figure 2:**
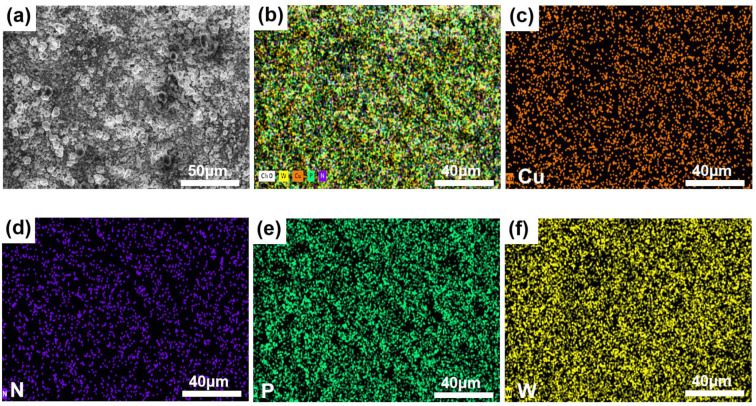
SEM of catalyst (**a**) and EDS of catalyst (**b**–**f**).

From the SEM (Figure 2a) of the catalyst, it can be seen that the catalyst has a loose porous and small particle crystal structure. In the EDS analysis diagram (Figure 2b–f), the following conclusions can be drawn: First, the catalyst contains Cu, N, P, and W. And then, Cu, N, P, and W are uniformly distributed in the catalyst.

**Figure 3:**
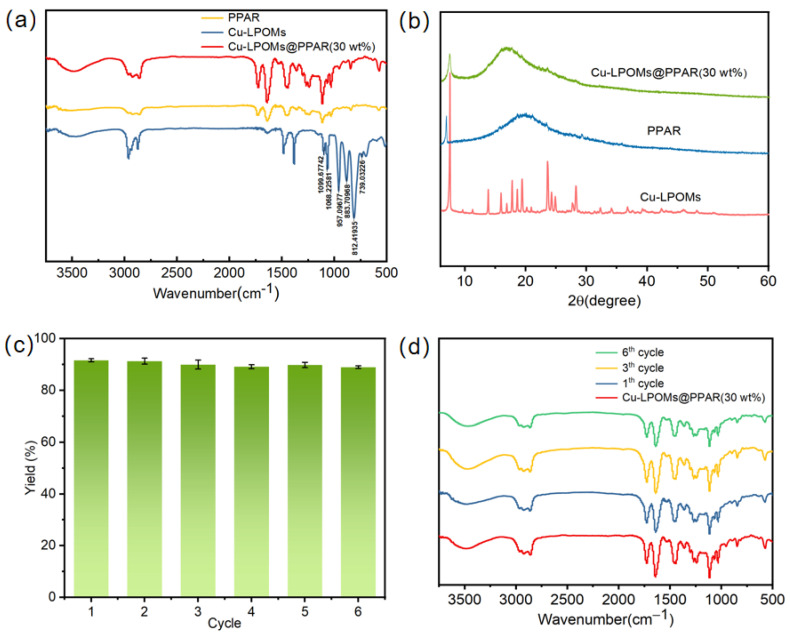
Characterization of Cu-LPOMs@PPAR (30 wt%). (**a**) The FT-IR of PPAR, Cu-LPOMs, Cu-LPOMs@PPAR (30 wt%). (**b**) The PXRD of PPAR, Cu-LPOMs, Cu-LPOMs@PPAR (30 wt%). (**c**) The recycling performance of Cu-LPOMs@PPAR (30 wt%). (**d**) The FT-IR spectra of six runs cycles.

The Fourier transform infrared (FT-IR) spectra of PPAR, Cu-LPOMs, and Cu-LPOMs@PPAR (30 wt%) exhibited characteristic peaks at 1100, 1060, 959, 886, 816, and 738 cm^−1^, which were attributed to Cu-LPOMs (Figure 3a) [43]. Additionally, the peaks of PPAR at 1728 and 1639 cm^−1^ were detectable as well as the representative Cu-O stretching (515 cm^−1^) in the spectra of Cu-LPOMs and Cu-LPOMs@CMC, further confirming the existence of Cu-LPOMs in Cu-LPOMs@PPAR. The PXRD patterns (Figure 3b) demonstrated that the sharp diffraction peaks of Cu-LPOMs were in agreement with previous reports, indicating high crystallinity and the good crystal quality of the synthesized catalyst [44]. The Cu-LPOMs@PPAR spectrum showed a similar peak position to PPAR, albeit with slight offset. Due to the successful encapsulation of Cu-LPOMs with photo polyacrylate resin, the peaks of Cu-LPOMs of Cu-LPOMs@PPAR (30 wt%) disappeared, demonstrating that Cu-LPOMs and PPAR were thoroughly mixed. Additionally, the UV-Vis absorption spectra of Cu-LPOMs@PPAR (30 wt%), PPAR, and Cu-LPOMs revealed distinct absorption peaks for Cu-LPOMs, providing evidence for the homogeneous mixing between Cu-LPOMs and PPAR (Figure S21).

Moreover, the stability and recyclability of Cu-LPOMs@PPAR (30 wt%) were investigated using benzyl alcohol as the substrate. The performance of Cu-LPOMs@PPAR (30 wt%) was assessed over six cycles, and the results demonstrated its sustained effectiveness even after multiple cycles, facilitating catalyst recovery and recycling (Figure 3c). Importantly, no catalyst debris was detected in the solution, indicating the inherent stability of the Cu-LPOMs@PPAR (30 wt%). Remarkably, the PXRD tests confirmed the well-preserved phase integrity of Cu-LPOMs@PPAR (30 wt%) throughout six consecutive recycling tests (Figure S22). Additionally, the FT-IR spectra of Cu-LPOMs@PPAR (30 wt%) exhibited negligible changes after six cycles, underscoring the catalyst’s exceptional structural stability and resistance to decomposition (Figure 3d). These findings further attest to the robustness and longevity of the Cu-LPOMs@PPAR (30 wt%) catalyst, thereby highlighting its potential for practical applications.

Additionally, the impact of catalyst loading on its performance was investigated. Employing Cu-LPOMs@PPAR with a loading of 10 wt% and 20 wt% resulted in product yields of 74% and 83%, respectively. Notably, the sample with 30 wt% and 40 wt% Cu-LPOMs loadings exhibited yields of 95% and 93%, indicating only a slight change with the increase in Cu-LPOMs loading (Figure S23). Hence, the optimal loading for Cu-POMs was determined to be 30 wt% for the oxidation of benzyl alcohol. Furthermore, it is worth mentioning that this loading achieved significantly high yields.

### 2.2. Electrocatalytic Property of Cu-LPOMs@PPAR

**Figure 4:**
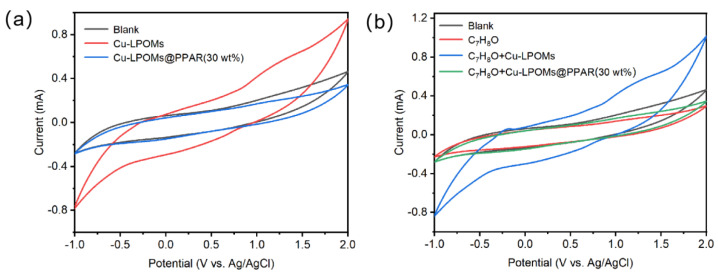
Electrochemical measurements of catalyst and reaction substrates using the carbon cloth as working electrode with a scan rate of 50 mV s^−1^. (**a**) CVs of Cu-POMs@PPAR (30 wt%) and Cu-LPOMs in acetonitrile containing n-Bu_4_NBF_4_ (0.007 g). (**b**) CVs of Cu-POMs@PPAR (30 wt%) and Cu-LPOMs in acetonitrile, conditions: n-Bu_4_NBF_4_ (0.007 g), C_7_H_8_O (0.25 mmol).

The electrocatalytic activity of Cu-LPOMs@PPAR (30 wt%) was originally assessed via cyclic voltammetry (CV) analysis (Figure 4). Figure 4a displays the cyclic voltammograms of Cu-LPOMs and Cu-LPOMs@PPAR (30 wt%) in the absence of the reaction substrate (0.007 g n-Bu_4_NBF_4_ serving as the electrolyte), revealing a lower current response for Cu-LPOMs@PPAR (30 wt%) compared to Cu-LPOMs. The incorporation of Cu-LPOMs@PPAR (30 wt%) demonstrates a rapid catalytic response towards electrochemical oxidation, exhibiting a lower oxidation potential than Cu-LPOMs (Figure 4b). Based on these findings, the porous structure of Cu-LPOMs@PPAR (30 wt%) is anticipated to serve as a promising electrocatalyst for subsequent indirect electrocatalytic reactions.

### 2.3. Selection of Experimental Conditions

Encouragingly, under constant voltage conditions (5 V) for 8 h, the oxidation of benzyl alcohol to benzaldehyde using a carbon anode and a carbon cathode with n-Bu_4_NBF_4_ as an electrolyte resulted in a high yield of the target product 1b (Table 1, entry 1). It is evident that without a catalyst, the yield of the product is low (50%) (Table 1, entry 2). Expectedly, when no electric current was passed through the system, the reaction did not occur (Table 1, entry 3). Furthermore, altering the voltage had a negative impact on product yields, with a decrease and increase in voltage resulting in 75% and 83% yields, respectively (Table 1, entries 4 and 6) compared to entry 5. Additionally, various solvents (CH_3_CN, H_2_O, and DMF) were evaluated, and none of the alternative reaction media exhibited superior performance to CH_3_CN (Table 1, entries 1, 7, and 8). It is worth noting that replacing n-Bu_4_NBF_4_ with n-Bu_4_NI led to a rapid reduction in the yield of benzaldehyde to 58% (Table 1, entry 9). Moreover, using a platinum plate as the cathode resulted in unsatisfactory results (Table 1, entry 10). Based on this, we hypothesized that n-Bu_4_NBF_4_ has a better ability for electrical conduct, and carbon has a better adsorption capacity, which is favorable for the reaction. Thus, the carbon surface, serving as the cathode, may be more suitable for the electrocatalytic oxidation of benzyl alcohol compared to other electrode materials.

### 2.4. Oxidant-Free Electrochemical Oxidative Benzyl Alcohols

Under the optimized conditions, we have investigated the scope and generality of this electrochemical oxidation reaction by examining a range of benzyl alcohol derivatives and aliphatic alcohol. The results are summarized in Table 2. By introducing various substituents (-Cl, -CH_3_, -OCH_3_, -NO_2_) at the para-position of the benzene ring, we were able to obtain the desired products (1b, 2b, 3b, 4b). The yield of benzyl aldehydes increased upon substitution with -CH_3_ and -OCH_3_, decreased upon substitution with -NO_2_, and remained relatively unchanged upon substitution with -Cl. Moreover, steric hindrance played a role, as evidenced by the lower yield of 3,5-dimethoxyacetophenone (5b) compared to its meta-counterpart. Additionally, when halogens were introduced at the ortho-position of benzyl alcohol, the yields of 7b, 8b, and 9b increased sequentially. Substituting the ortho and para positions of benzyl alcohol with methyl groups resulted in high yields for products 2b and 6b, respectively. Interestingly, Table 2 entry 10 demonstrates that a non-aromatic system yields better oxidation results.

The electron-donating groups on benzyl alcohol contribute to the enhanced oxidation yield through ortho-para activation. Furthermore, the yield is influenced by the electron-supplying capacity of the substituents, with higher yields observed for reactants with stronger electron-donating abilities. Conversely, bulky substituents hinder contact with the catalyst, leading to lower yields. In the meantime, we investigated the oxidation of cyclohexanol, which demonstrated promising yields for both aromatic and non-aromatic alcohols.

**Figure 5:**
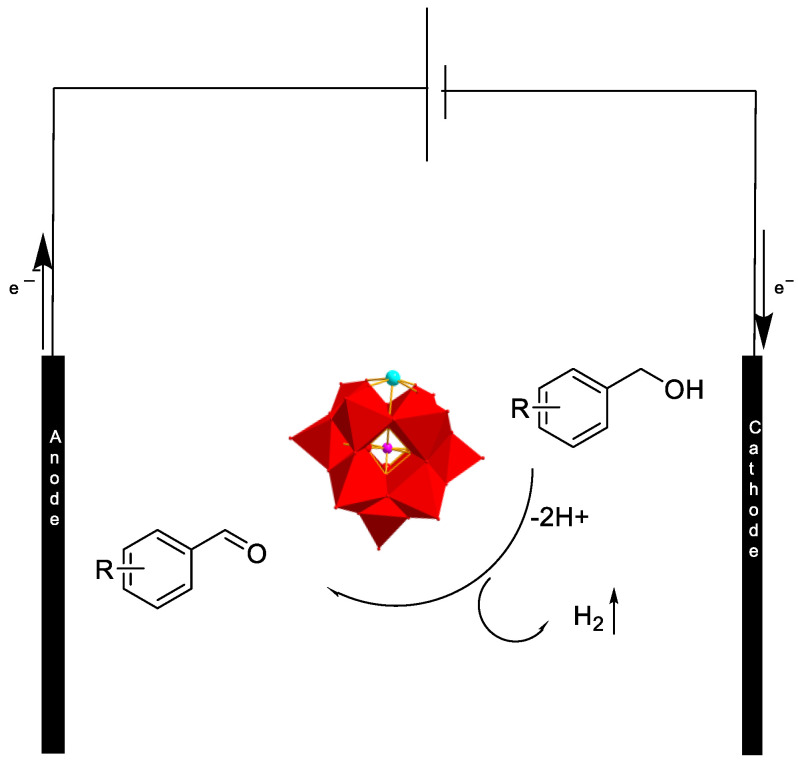
The oxidation mechanism of benzyl alcohol.

The mechanism underlying the electrocatalytic oxidation of benzyl alcohol to benzaldehyde involves the initial adsorption of benzyl alcohol onto the Cu-LPOMs@PPAR catalyst (Figure 5). Subsequently, benzyl alcohol undergoes oxidation on the carbon electrode, leading to the production of hydrogen as a byproduct. Ultimately, benzaldehyde is obtained at the anode.

## 3. Materials and Methods

### 3.1. Materials

Unless otherwise noted, materials were obtained from commercial suppliers and used without further purification. The reagents and chemicals, including sodium tungstate dihydrate (99.5%), sodium phosphate dibasic dihydrate (98%), copper sulfate pentahydrate (AR), photo polyacrylate resin and tetrabutylammonium bromide (TBAB) (99.0%), tetrabutylammonium tetrafluoroborate (*n*-Bu4NBF4), MOLEGRID^TM^ WASHABLE photopolymer resin, etc., were all purchased from Energy Chemical.

### 3.2. Characterization

The X-ray diffraction (PXRD) patterns were recorded on a Rigaku Smartlab3 X-ray Powder Diffractometer equipped with a Cu sealed tube (λ = 1.54178 Å) in the range of 5° to 50° at room temperature. The Fourier transform infrared spectrometry (FT-IR) analyses were measured on a Nicolet 5700 spectrophotometer in the range 400–4000 cm^−1^. The morphology and microstructure of the samples were observed using scanning electron microscopy (SEM), Thermo Fisher Scientific FIB-SEM GX4. The UV-Vis spectroscopy was measured using UV-2600. Column chromatography was hand packed with silica gel or aluminum oxide (200–300 mesh). The quantity of hydrogen evolved was determined using a Techcomp GC-2030 gas chromatograph with a 5 Å molecular sieve column (2 m × 2 mm) and a thermal conductivity detector (TCD). After the reaction was completed, the resulting mixture was finally analyzed using Waters E2695 high performance liquid chromatography (HPLC) with naphthalene as an internal standard; the column model is Bridge C18 5 µm, 4.6 mm × 250 mm and the detector for HPLC is Waters 2998 PDA detector. High-resolution mass spectra (HRMS) were recorded on a UPLC I-CLASS/XEVO G2-XS QTOF (ESI). TLC was carried out with 0.2 mm thick silica gel plates (GF254). Visualization was accomplished by UV light. The instrument used for electrolysis is ElectraSyn 2.0 (made in America), and the Carbon plate (53 mm × 8 mm × 1.5 mm) was purchased from Aika (Guangzhou, China) instrument equipment Co., Ltd.

### 3.3. Synthesis of (TBA)_4_H[PW_11_CuO_39_](Cu-LPOMs)

Precursors, including sodium tungstate dihydrate (10 mmol), sodium phosphate dibasic dihydrate (0.92 mmol), and copper sulfate pentahydrate (1.2 mmol), were dissolved in 20 mL of water, and the pH of the solution was adjusted to 4.8 using a 1.0 M HNO_3_ solution. The resulting solution was heated to a temperature range of 80 °C to 85 °C under continuous stirring. Simultaneously, TBA (4.5 mmol) was dissolved in 5 mL of water and added dropwise to the aforementioned mixture, leading to the formation of a light blue solution. Subsequently, the solution was slowly cooled to room temperature, resulting in the formation of a precipitate denoted as (TBA)_4_H[PW_11_CuO_39_]. The precipitate was then subjected to filtration and recrystallization using a mixture of acetonitrile and water. Finally, the blue precipitates were dried under vacuum at 100 °C, resulting in the formation of (TBA)_4_H[PW_11_CuO_39_], which was identified and referred to as Cu-LPOMs.

### 3.4. Synthesis of Cu-LPOMs@PPAR

A mixture of Cu-LPOMs (20 g) and photo polyacrylate resin (250 g) was placed into a 50 mL beaker, followed by thorough stirring for 5 h in a dark environment at a temperature of 40 °C to prevent exposure to light. Prior to printing, the 3D model file intended for printing was imported into the printer’s software (Polydevs version number: 3.0.6.47) and certain essential settings, such as support structures and placement orientation, were adjusted. Once the settings were finalized, the file was sliced and saved onto the printer’s USB drive. Subsequently, the photo polyacrylate resin blended with Cu-LPOMs was loaded into the printer’s cartridge. The cassette, along with the platform, was secured firmly in place. The USB drive was inserted into the printer and the printer was powered on. Through the printer’s control panel, the desired file was selected, and additional parameters such as exposure power and duration were set accordingly. Upon pressing the Print button, the printer commenced operation. Following the completion of the printing process, the printed model was carefully removed from the printer’s platform, and subsequent post-processing steps were performed, including cleaning with 95-degree alcohol, drying, and post-curing. Ultimately, a fully formed Cu-LPOMs@PPAR structure was obtained.

### 3.5. Procedure for Electrocatalytic Oxidation of Benzyl Alcohols by Cu-LPOMs@PPAR

Benzyl alcohol (0.25 mmol), n-Bu_4_NBF_4_ (7 mg), CH_3_CN (3 mL), and a piece of Cu-LPOMs@PPAR were put in a dried ElectraSyn 2.0 (Figure S24) vial equipped with a stir bar. The tube was equipped with a carbon plate (15 mm × 15 mm × 0.33 mm) serving as both the anode and cathode. The reaction mixture was stirred and subjected to electrolysis at a constant voltage of 5 V, under ambient temperature, for a duration of 8 h. Following the completion of the reaction, the mixture was cooled to room temperature, and the product was purified using flash chromatography over neutral alumina, utilizing a petrol/ethyl acetate eluent gradient (ranging from 100:1 to 50:1).

### 3.6. Electrochemical Measurements

In the experimental setup, the Pt foil was employed as the counter electrode, whereas the Ag/AgCl electrode functioned as the reference electrode. The working electrode consisted of carbon cloth material. Chronoamperometry investigations were carried out at ambient temperature utilizing an electrochemical workstation (SP-150, Bio-Logic, Seyssinet-Pariset, France) equipped with a conventional three-electrode configuration. Cyclic voltammetry (CV) measurements involved subjecting the catalyst and substrate to a potential window spanning from −1 V to 2 V (vs. Ag/AgCl), with scan rates ranging from 50 mV s^−1^.

## 4. Conclusions

In conclusion, we successfully synthesized copper monosubstituted phosphotungstate-based polyacrylate resins (Cu-LPOMs@PPAR) with immobilization and recyclability using 3D printing technology, which exhibit exceptional properties such as a porous and loose architecture, remarkable stability, well-dispersed Cu-LPOMs, shape designability, and adjustable loadings of Cu-LPOMs. Noteworthy, the Cu-LPOMs@PPAR catalyst demonstrates excellent performance in the electrocatalytic oxidation of various benzyl alcohol derivatives, yielding the corresponding aldehydes in moderate-to-excellent yields. Remarkably, it can be easily separated and reutilized for six cycles without suffering significantly reduced catalytic activity. These insightful investigations provide a rational foundation for the development of Cu-LPOMs@PPAR as an efficient electrocatalyst for electrochemical oxidation, leading to the production of value-added chemicals with promising potential for future industrial applications.

## Figures and Tables

**Table 1:** Optimization of the reaction conditions ^a^.

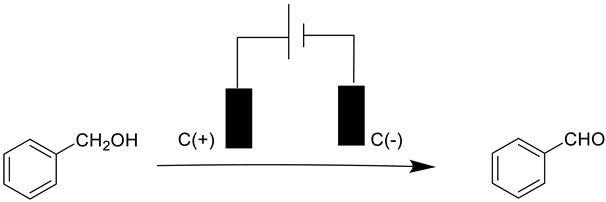			
1a		1b	
**Entry**	**Variation(s) from the Standard Conditions**		**Yield ^b^ (%)**
1	None		95
2	No catalyst		50
3	Without electricity		n.d.
4	3 V, 8 h		75
5	5 V, 8 h		90
6	7 V, 8 h		83
7	H_2_O as solvent		n.d.
8	DMF as solvent		n.d.
9	n-Bu_4_NI instead of n-Bu_4_NBF_4_		58
10	Platinum plate as the cathode		65

^a^ standard conditions: carbon plate (15 mm × 15 mm × 0.33 mm) as anode, carbon plate (15 mm × 15 mm × 0.33 mm) as cathode, constant voltage = 5 V, benzyl alcohols (0.25 mmol), n-Bu_4_NBF_4_ (7 mg), CH_3_CN (3.0 mL), r. t., under air atmosphere, 8 h. ^b^ Yields were determined by GC with C_6_H_5_Cl as an internal standard and confirmed by GC-MS.

**Table 2:** The scope of electrocatalysis oxidation of benzyl alcohol ^a^.

Entry	Benzyl Alcohol (a)	Aldehyde (b)	Yield ^b^ (%)
0	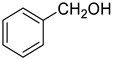	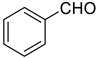	75
1	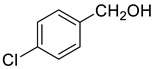	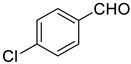	85
2	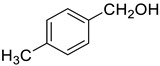	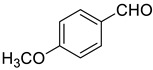	91
3	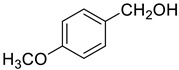	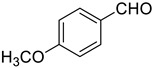	90
4	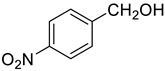	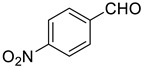	80
5	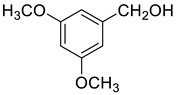	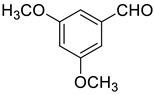	75
6	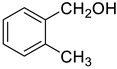	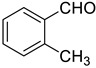	92
7	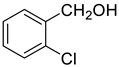	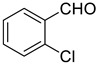	80
8	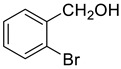	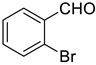	86
9	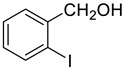	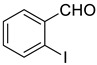	90
10	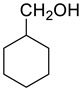	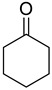	87

^a^ Reaction conditions: carbon _plate_ (15 mm × 15 mm × 0.33 mm) as anode, carbon _plate_ (15 mm × 15 mm × 0.33 mm) as cathode, constant voltage = 5 V, benzyl alcohols (0.25 mmol), TBEA (7 mg), CH_3_CN (3.0 mL), r. t., under air atmosphere, 15 h. ^b^ Yields were determined by GC and confirmed by GC-MS. c:12 h.

## Data Availability

The data used to support the findings of this study are available from the corresponding author upon request.

## Supplementary Materials

The following supporting information can be downloaded at: https://www.mdpi.com/article/10.3390/molecules28186460/s1.
