# Supplementary material for: Oxidant-Free Electrochemical Direct Oxidative Benzyl Alcohols to Benzyl Aldehydes Using Three-Dimensional Printing PPAR Polyoxometalate
Source: Molecules. 2023 Sep 6;28(18):6460. doi: 10.3390/molecules28186460 (PMC10534777; doi:10.3390/molecules28186460)
Supplement: Supplementary file 1 [file molecules-28-06460-s001.zip › molecules-2594864-supplementary.pdf]

Oxidant-Free Electrochemical Direct Oxidative Benzyl Alcohols to Benzyl Aldehydes Using Three-Dimensional Printing PPAR Polyoxometalate

Wenhui Zhang [1], Ran Liu [1], Xueyan Lv [2], Lirong Jiang [1], Silu Tang [1], Gang Liu [1], Guodong Shen [1], Xianqiang Huang [1], Chen Ma [2,*] and Bingchuan Yang [1,2,3,*]

[1] School of Chemistry and Chemical Engineering, Liaocheng University, Liaocheng 252000, China; zwh2963105101@163.com (W.Z.); liuranbjt@163.com (R.L.); jlr17861826211@163.com (L.J.); tsl2766560274@163.com (S.T.); shenguodong@lcu.edu.cn (G.S.); hxq@lcu.edu.cn (X.H.)

[2] School of Chemistry and Chemical Engineering, Shandong University, Jinan 250100, China; lxy17852267584@163.com

[3] College of Chemistry and Chemical Engineering, Qilu Normal University, Jinan 250013, China

* Correspondence: chenma@sdu.edu.cn (C.M.); yangbingchuan@lcu.edu.cn (B.Y.)

Characterizations and instruments

The X-ray diffraction (PXRD) patterns were recorded on a Rigaku Smartlab3 X-ray Powder Diffractometer equipped with a Cu sealed tube (λ = 1.54178 Å) in the range of 5° to 50° at room temperature. The Fourier transform infrared spectrometry (FT-IR) analyses were measured on a Nicolet 5700 spectrophotometer in the range 400-4000 cm^{-1}. The morphology and microstructure of the samples were observed by scanning electron microscopy (SEM), Thermo Fisher Scientific FIB-SEM GX4. The UV-Vis spectroscopy was measured on UV-2600. Column chromatography was hand packed with silica gel or aluminum oxide (200-300 mesh). The quantity of hydrogen evolved was determined using a Techcomp GC-2030 gas chromatograph with a 5 Å molecular sieve column (2 m × 2 mm) and a thermal conductivity detector (TCD). After the reaction was completed, the resulting mixture was finally analyzed by Waters E2695 high performance liquid chromatography (HPLC) with naphthalene as an internal standard, the column model is Bridge C18 5 μm, 4.6 mm × 250 mm and the detector for HPLC is Waters 2998 PDA detector. High-resolution mass spectra (HRMS) were recorded on a UPLC I-CLASS/XEVO G2-XS QTOF (ESI). TLC was carried out with 0.2 mm thick silica gel plates (GF254). Visualization was accomplished by UV light. The instrument for electrolysis is ElectraSyn 2.0 (made in America), the Carbon plate (53 mm × 8 mm × 1.5 mm) was purchased from Aika (Guangzhou, China) instrument equipment Co., LTD.

1. **The GC-MS analysis of products**

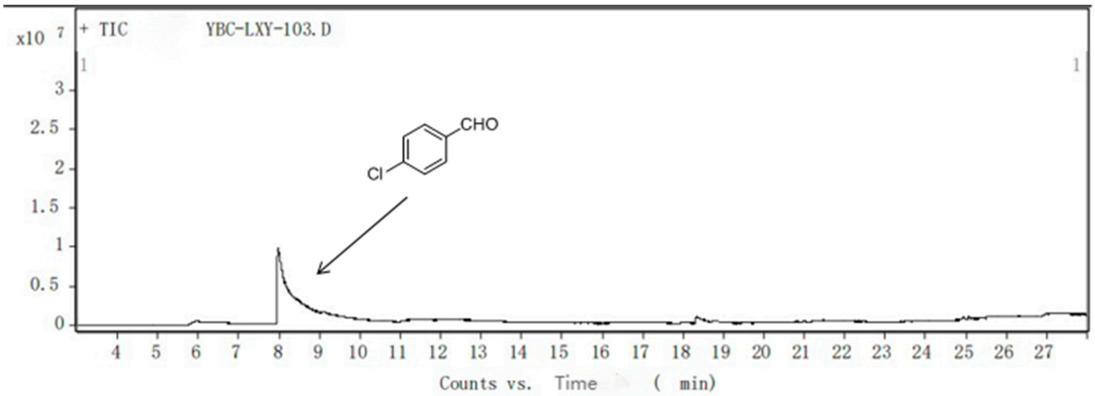

Figure S1. Gas chromatogram of product 1b.

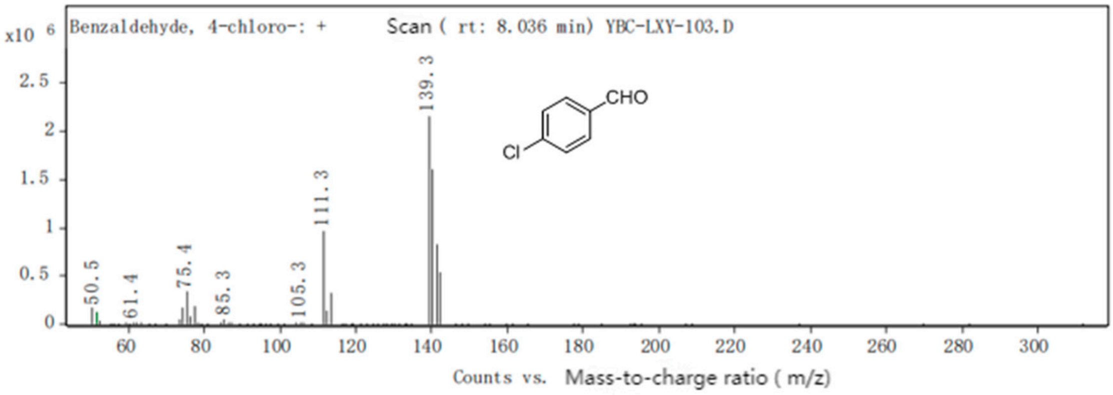

Figure S2. The mass spectrum of product 1b.

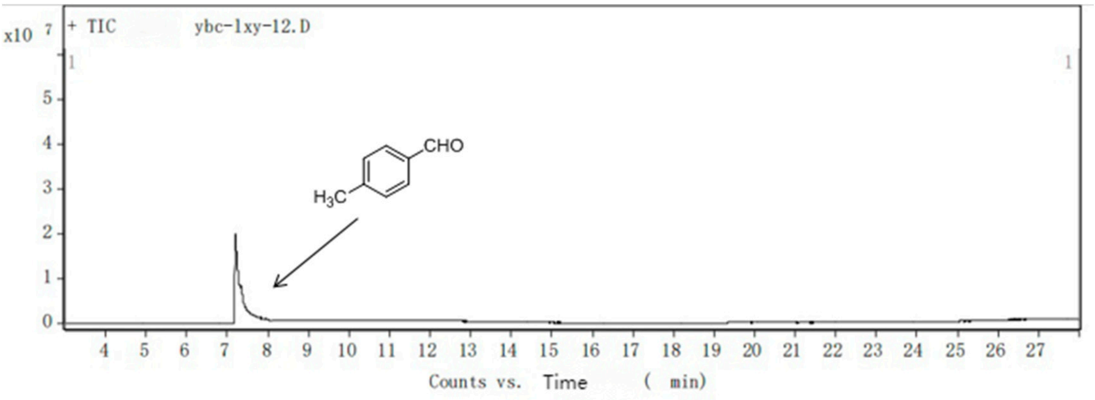

Figure S3. Gas chromatogram of product 2b.

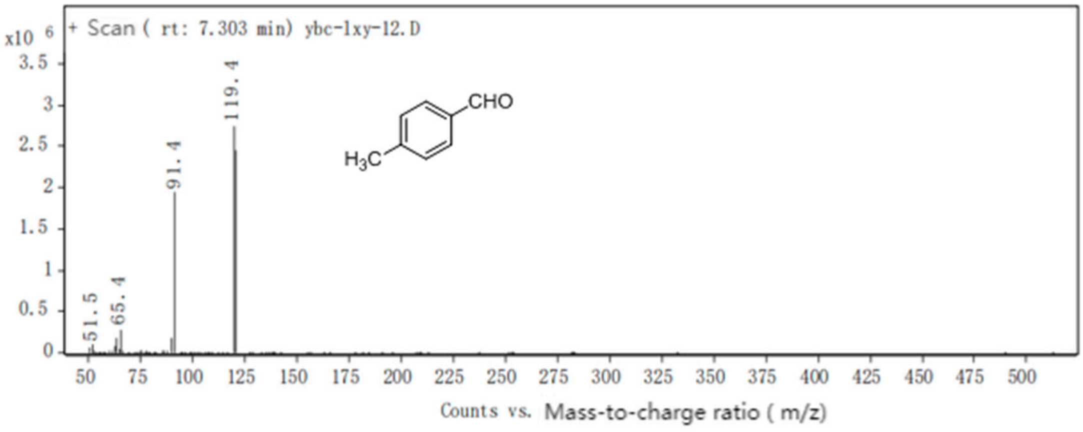

Figure S4. The mass spectrum of product 2b.

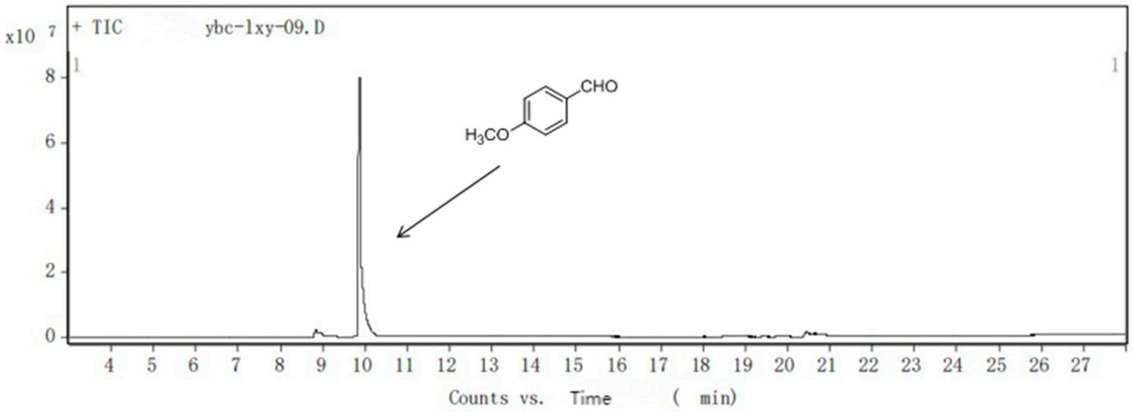

Figure S5. Gas chromatogram of product 3b.

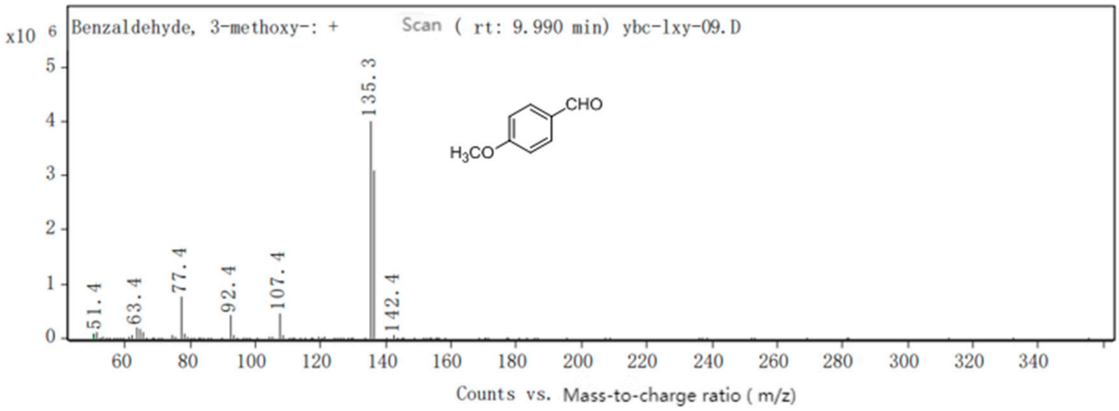

Figure S6. The mass spectrum of product 3b.

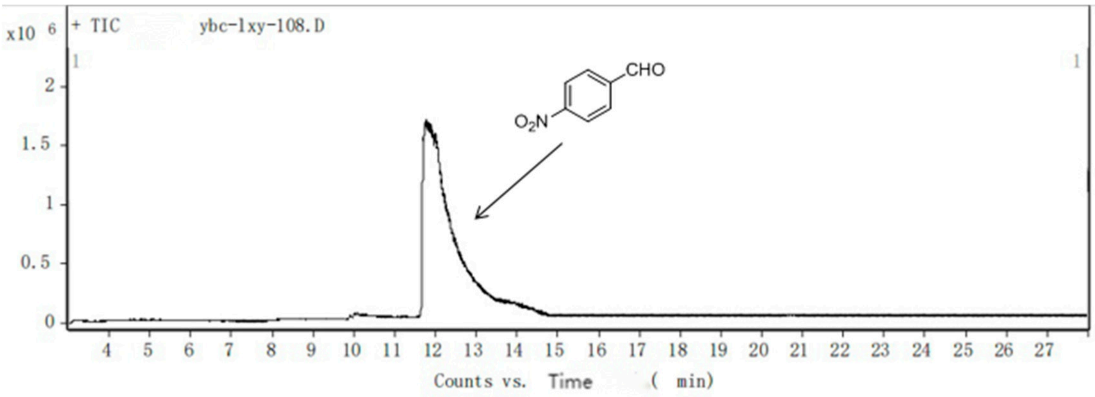

Figure S7. Gas chromatogram of product 4b.

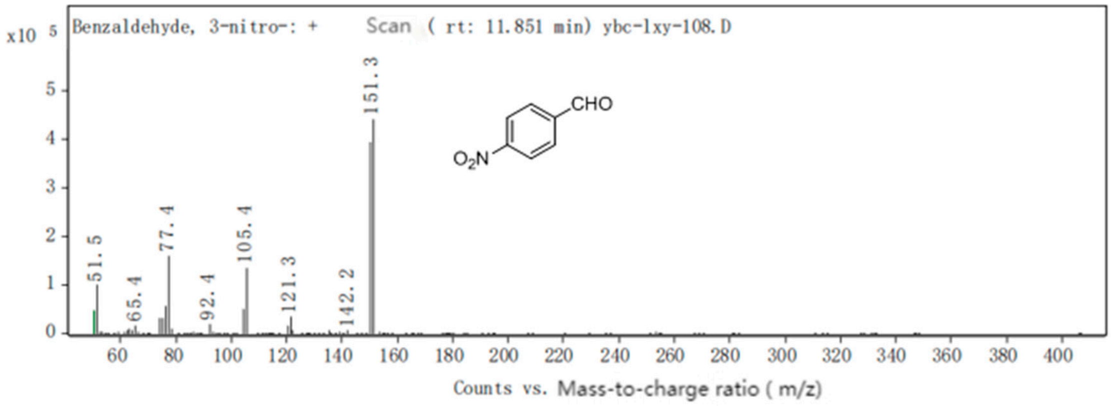

Figure S8. The mass spectrum of product 4b.

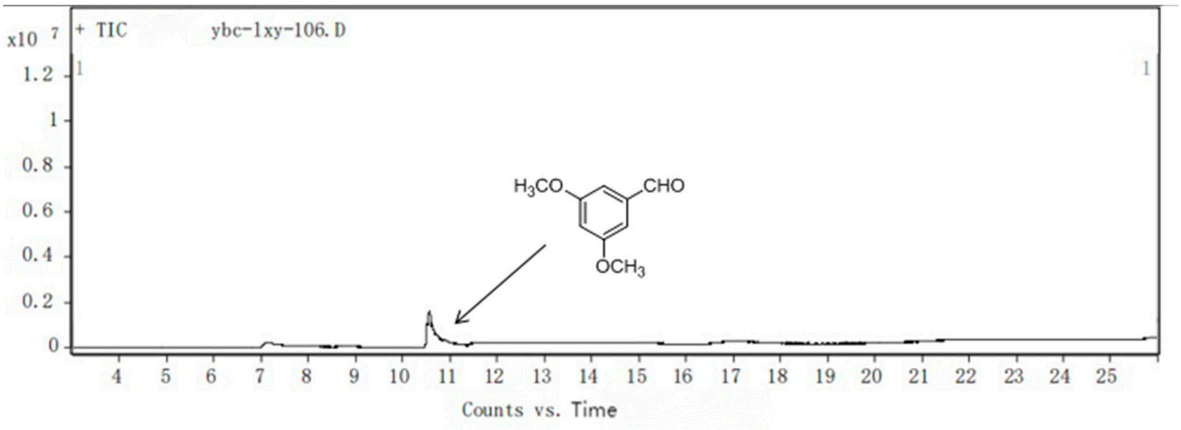

Figure S9. Gas chromatogram of product 5b.

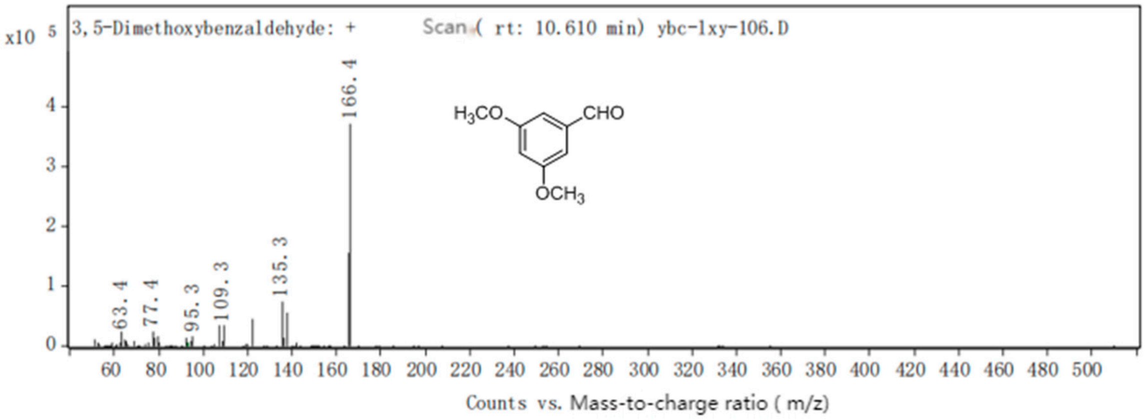

Figure S10. The mass spectrum of product 5b.

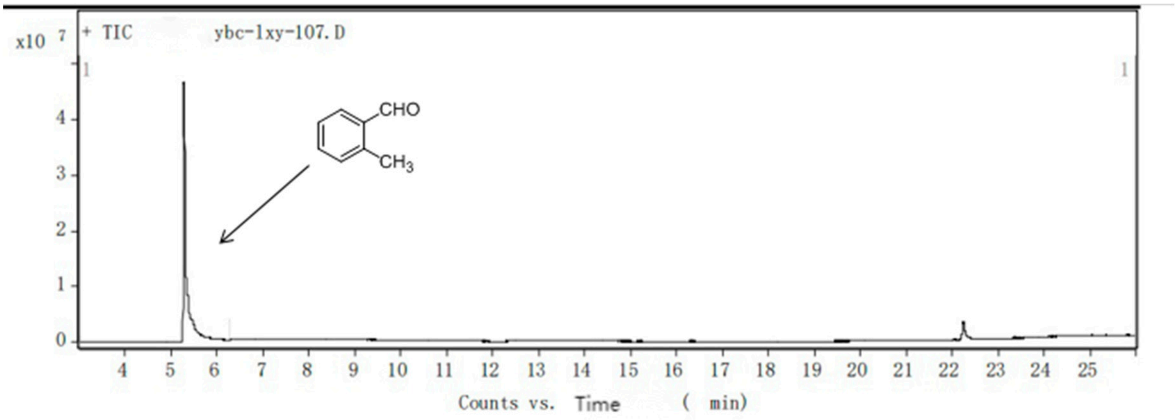

Figure S11. Gas chromatogram of product 6b.

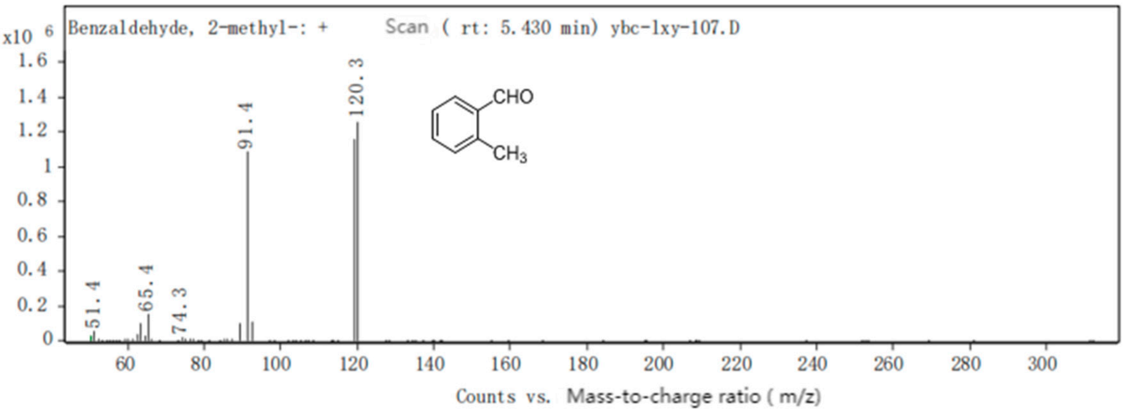

Figure S12. The mass spectrum of product 6b.

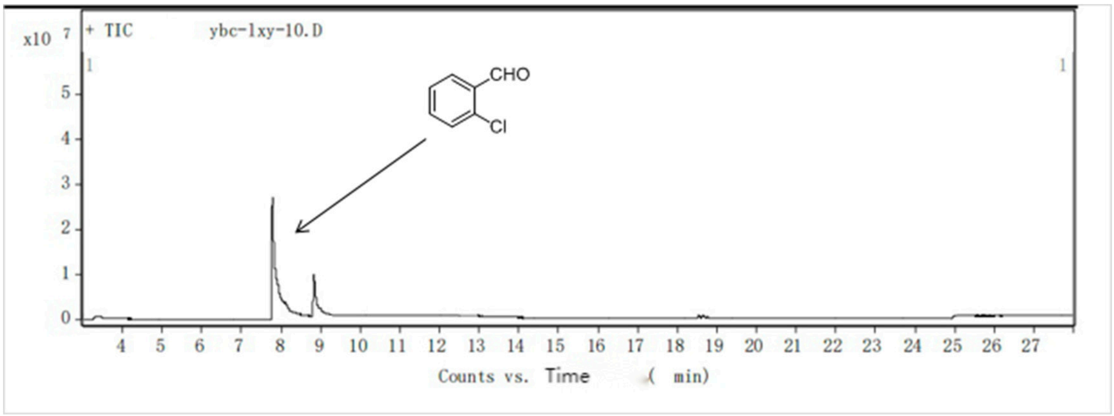

Figure S13. Gas chromatogram of product 7b.

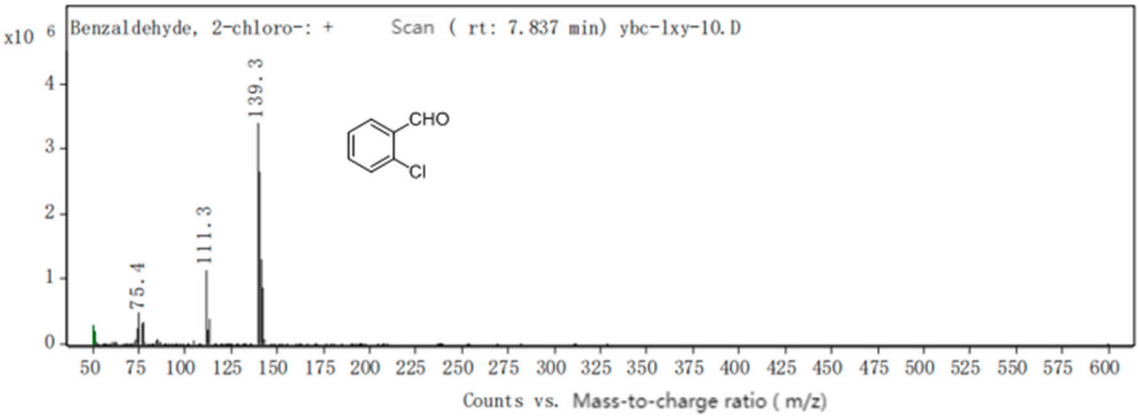

Figure S14. The mass spectrum of product 7b.

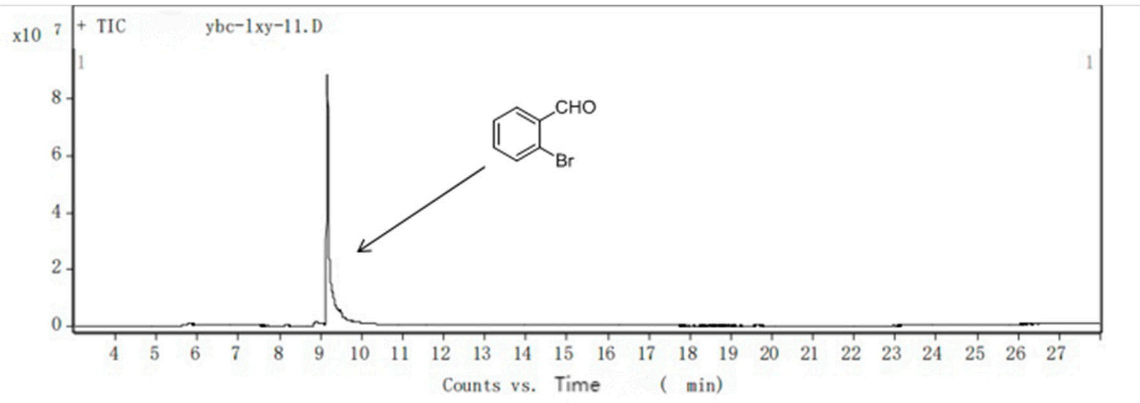

Figure S15. Gas chromatogram of product 8b.

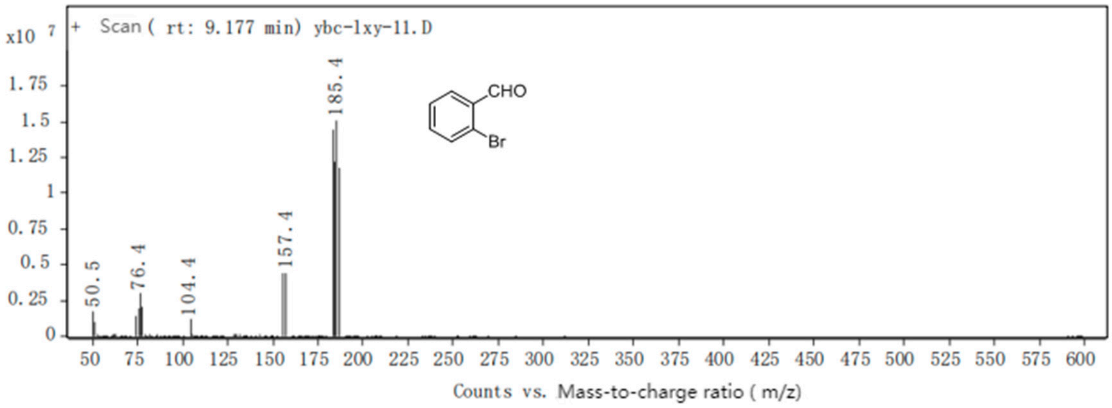

Figure S16. The mass spectrum of product 8b.

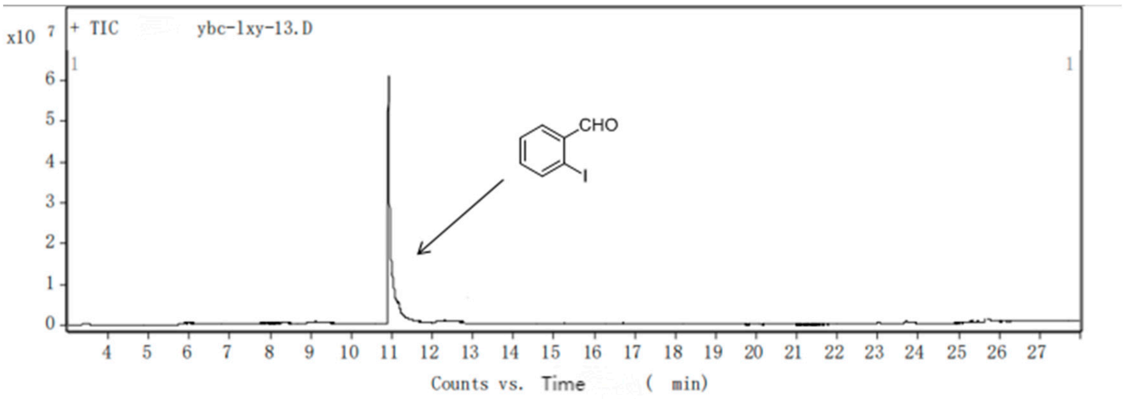

Figure S17. Gas chromatogram of product 9b.

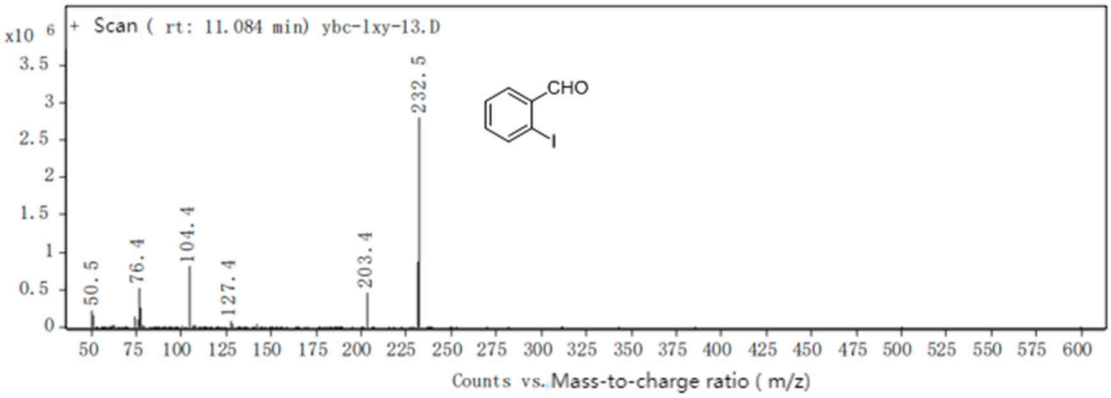

Figure S18. The mass spectrum of product 9b.

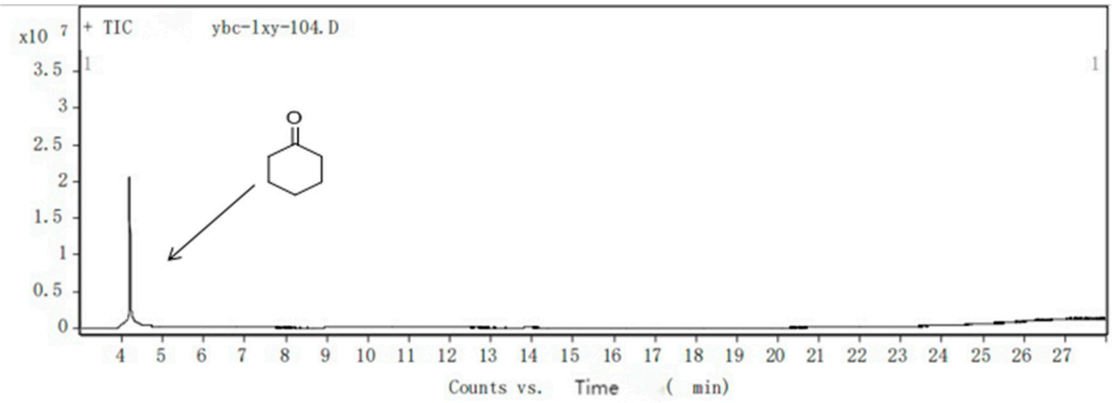

Figure S19. Gas chromatogram of product 10b.

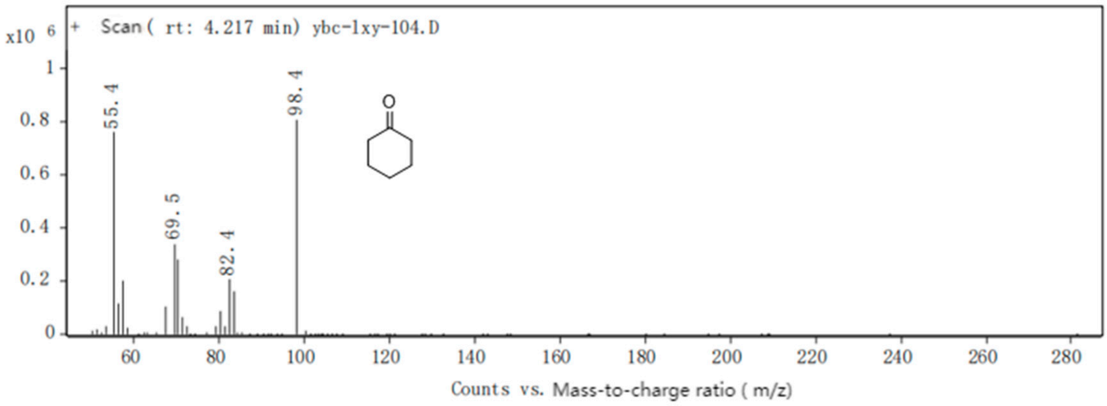

Figure S20. The mass spectrum of product 10b.

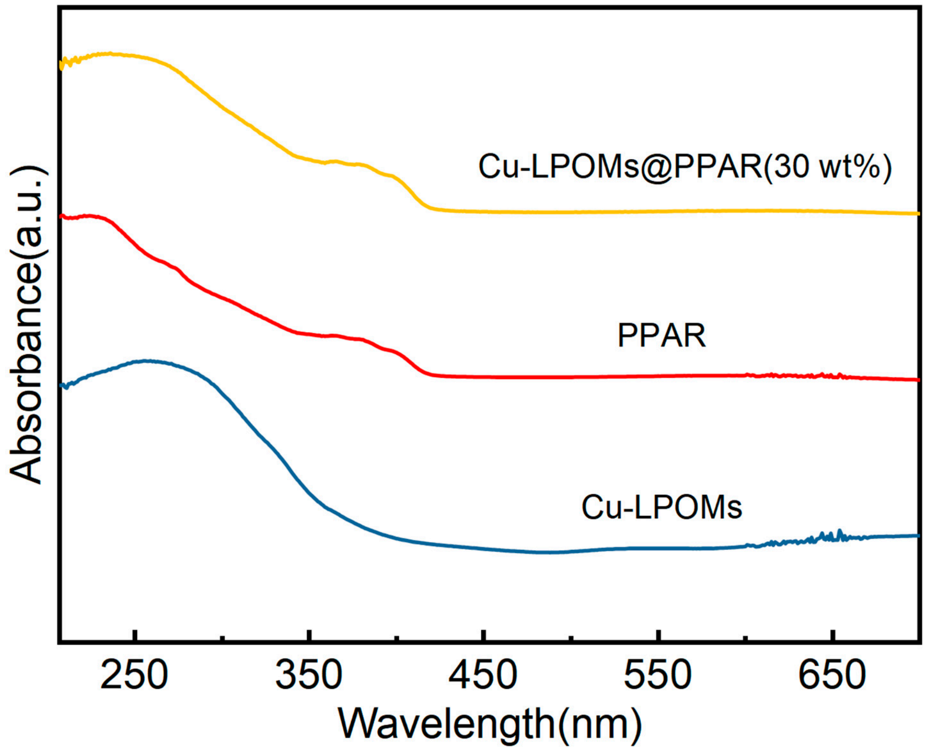

Figure S21. UV-Vis absorption spectra of Cu-LPOMs@PPAR (30 wt%), PPAR, and Cu-LPOMs.

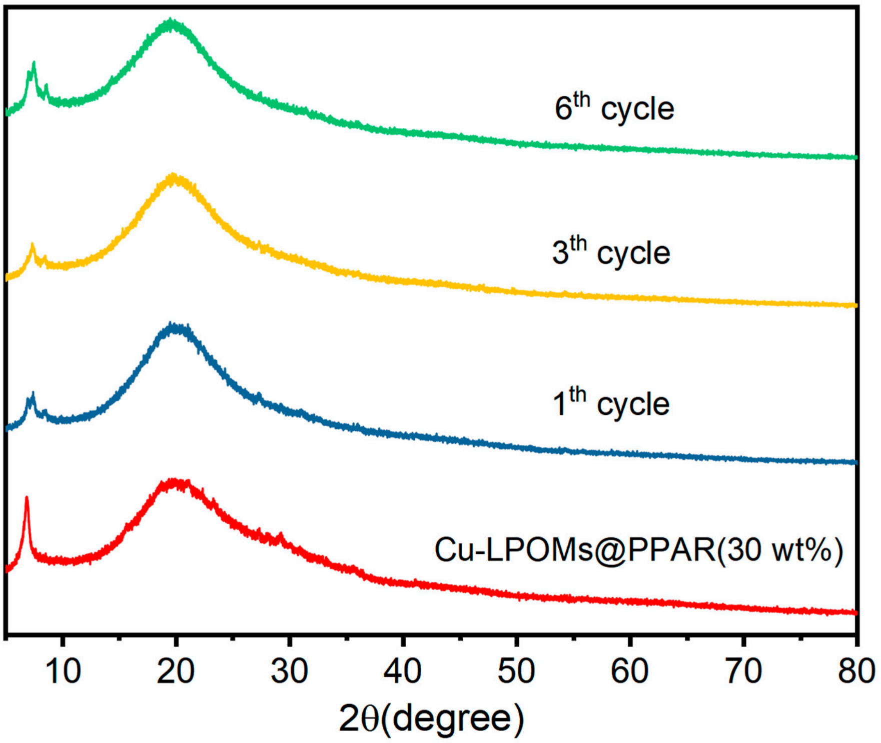

Figure S22. PXRD patterns of Cu-LPOMs@PPAR (30 wt%) after recycling tests.

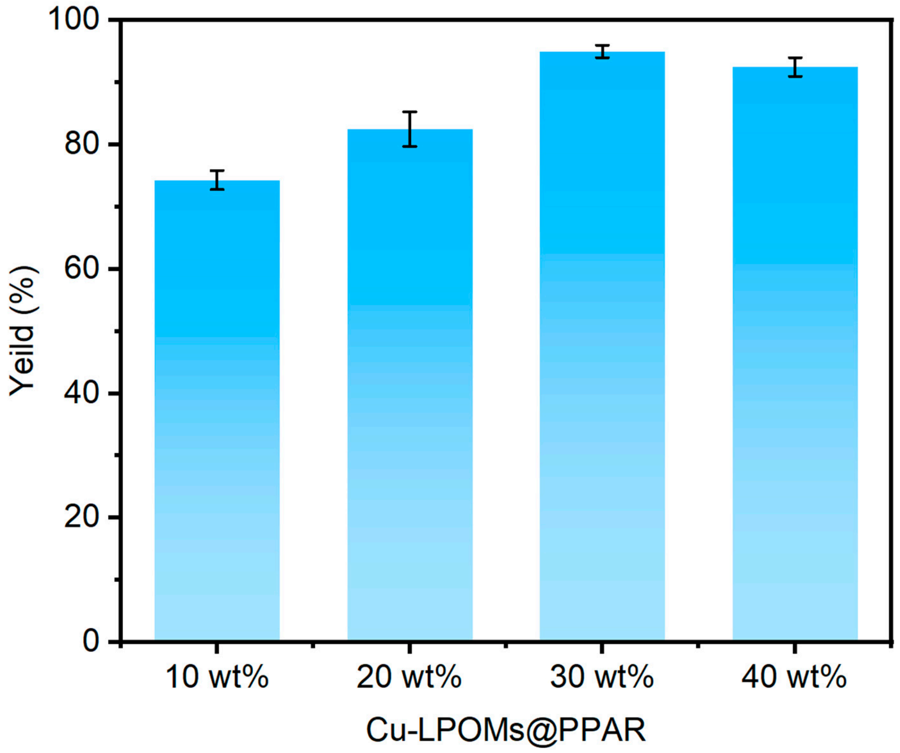

Figure S23. The recycling experiment results on the electrosynthesis of CBS catalyzed by Cu-LPOMs@PPAR (30 wt%).

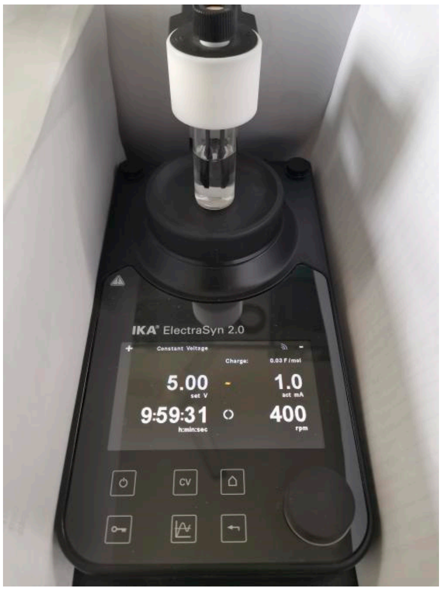

Figure S24. The picture of electorcatalytic reactor.
